# Data transmission reduction formalization for cloud offloading-based IoT systems

**DOI:** 10.1186/s13677-023-00424-8

**Published:** 2023-03-28

**Authors:** Aya Elouali, Higinio Mora Mora, Francisco José Mora-Gimeno

**Affiliations:** Department of Computer Science Technology and Computation, University of Alicante, Alicante, Spain

**Keywords:** Data transmission reduction, Cloud computing, IoT cameras, Data offloading

## Abstract

Computation offloading is the solution for IoT devices of limited resources and high-cost processing requirements. However, the network related issues such as latency and bandwidth consumption need to be considered. Data transmission reduction is one of the solutions aiming to solve network related problems by reducing the amount of data transmitted. In this paper, we propose a generalized formal data transmission reduction model independent of the system and the data type. This formalization is based on two main ideas: 1) Not sending data until a significant change occurs, 2) Sending a lighter size entity permitting the cloud to deduct the data captured by the IoT device without actually receiving it. This paper includes the mathematical representation of the model, general evaluation metrics formulas as well as detailed projections on real world use cases.

## Introduction

In today’s world, IoT systems are more and more overwhelming. All electronic devices are becoming connected. From lamps and refrigerators in smart homes, smoke detectors and cameras for monitoring systems, to scales and thermometers for healthcare systems until phones, cars and watches in smart cities. All these connected devices generate a huge amount of data collected from the environment. International Data Corporation (IDC) estimates that by 2025 there will be 55*.*9 billion connected devices worldwide generating 79*.*4ZB of data [1]. To take advantage of these data, a processing phase is needed in order to extract useful information allowing a best management of the system [2].

Computation offloading is a mechanism used in IoT systems, it consists of outsourcing the data processing task to a more powerful machine [3, 4]. It is generally performed from an IoT device with limited capacities to a cloud server in order to benefit from its high storage and processing capacities [5]. However, the cloud server is geographically remote from the connected device which leads to a long communication delay and harms the effectiveness of the system [6]. Moreover, due to the incredibly increasing number of IoT devices and therefore offloading operations, the load on the network has increased significantly [7]. Especially that current IoT systems rely on LPWAN (Low Power Wide Area Network) for communication because of its low transmission power, however, this kind of network suffers from considerable limitations in bandwidth [8]. The type of data manipulated also plays a very important role, voluminous and heavy data such as the images and videos are bandwidth consuming to a large extent. In most of current systems, connected objects capture data each time period and send it entirely to the cloud. However, to reduce network related problems it is becoming increasingly common to reduce the amount of data sent to the cloud, this concept is known as Data Transmission Reduction [9, 10]. Generally, data transmission reduction solutions rely on compression, prediction or classification methods. However, those methods suffer from several disadvantages such as data quality deterioration, prediction and classification errors, as well as the large processing performed at the IoT object’s side. Furthermore, those solutions are designed to measure for a precise problem which make it almost impossible to generalize the conceptual rules or to propose improvements for groups of systems or even formalize the evaluation methods.

In this paper, we propose a generalized model to formalize the reduction of latency and bandwidth consumption as well as the device’s resources consumption for IoT systems offloading data to the cloud. This generalized model do not depend on the IoT system or the data type and do not conduct heavy processing or cause a deterioration in the quality of data. It is based on two main ideas:

- Not sending data to the cloud until a meaningful change between the new captured and the previous one occurs.
- Once a change is detected, send to the cloud an entity (lighter than the captured data) that has been calculated on the device’s side. This entity is a relationship between the new captured data and its predecessor allowing the cloud server to calculate the captured data without really receiving it.

The main contribution of this work is generalizing and formalizing the data transmission reduction process without relying on any of the heavy methods sited previously. The proposed mathematically defined model permits its projection on different IoT systems. We also define generalized evaluation metrics to evaluate each projection. This paper is a more in-depth study of our previous paper [11] where we briefly explained the general idea of this model and it is organized as follows: in "Section 2: related work" section we review most relevant works in terms of proposing solutions to improve the functioning of IoT systems and we determine how our work is situated compared to them. Section 3: data transmission reduction model section presents the proposed solution, its components as well as generalized evaluation formulas. In "Section 4: use cases" section, we project this solution on real life data transmission systems while "Section 5: conclusions" section presents the conclusions of this proposal.

### Section 2: related work

In the literature, several solutions improving the performance of IoT systems offloading data have been proposed in different application tracks. We grouped works improving IoT systems’ performance into five categories according to the part of the system they are trying to enhance: the network, the processing, the storage, the quality of data and reducing the amount of data sent via the network.

In the first category, researchers are trying to improve the quality of the network. Authors of [12] proposed AI solutions to different network problems faced in different IoT systems, such as the random-access challenge to which they proposed a Deep Reinforcement Learning (DRL) method to help IoT devices making decisions about the number of random access resource blocks used in one random access. To the spectrum access and spectrum sensing challenges they suggested a Recurrent Neural Network (RNN) and a Neural Network Based on reservoir computing (RC) respectively. To solve the transmission parameters adjustment issue, they proposed to use a DRL model. While in [13], authors proposed a self-organization method to improve communications between IoT devices in a network. When an IoT device wants to send a packet to another one, a path comparison table is created containing coefficients characterizing each path. This coefficient is calculated by taking in consideration parameters such as energy, speed for the devices between the source and destination. Then, the optimal path is chosen to transmit the packet. In order to organize Wireless Sensor Networks (WSN) and preserve the network from hotspot problems, Baniata et al. proposed a protocol that aims to select appropriate cluster heads (node that collect data from cluster’s nodes and send it to the central station), create hybrid clusters in an optimal way, choose optimum route between cluster heads and the central station, and define the most suitable communication technology for each node [14]. In [15] the solution consisted on distributing the processing between three layers: connected devices in a smart city, an intermediate layer whose goal is to conduct computation, called a cloudlet, and the centralized cloud server. By distributing the processing tasks, the charge decreased and network related issues were greatly reduced.

In the second category, solutions were proposed to improve the processing part of the IoT system. In [16], authors suggested creating the AI model in the cloud benefiting from its computing capacities. Then send this model to the edge of the network so that the inference can be done near the device collecting data. In the same context, Koesdwiady et al. proposed to enhance the AI part of a traffic prediction system by adding weather data. They fusioned weather and traffic data at decision level. This was done by inserting the output of two Deep Belief Network (DBN) (one of them predicting traffic using traffic history and the other using current weather data) in another DBN predicting traffic based on both traffic history and the weather [17].

The third category presents solutions improving the storage in IoT devices as in [18]. In this paper, the authors proposed a hybrid Dynamic Random Access Memories (DRAM)-Phase Change Memories (PCM) in order to benefit from the advantages of each one of them and fit the requirements of IoT devices. The solution consists of storing the data in PCM memory then migrating the rows accessed by write operations much to the DRAM memory. In [19] using the blockchain technology, authors were able to improve the security of IoT data in untrusted storage as well as reducing the storage load of the system. However, the proposed solution was time-consuming which may cause problems for some IoT systems. In [20], authors proposed a Selective Memory Balancing (SMB) to improve the storage in IoT devices. They used a collaborative technique between the Energy Management Unit, smart devices and appliances in smart homes based on temporal observation.

Works improving the quality of data sent such as [8] are part of the fourth category. This paper proposed to use a CNN (Convolutional Neural Network) to compress image data before sending it to the cloud. This solution is most suitable for IoT devices and preserves the quality of the image better than traditional compression methods such as JPEG. In [21], authors proposed an IoT system architecture using blockchain for decentralized data management. This combination permits the system to have better management of breaks down as well as better security and trust since data is encrypted.

The last category concerns works proposing solutions to reduce the amount of data sent via the network. Solutions in this field can be classified into four categories depending on the method used: based on a prediction model, based on a classifier, based on compression and based on heuristic methods.

#### Based on a prediction model

Works based on prediction models rely on the use of a trained prediction model on both sides and only transmit poorly predicted values. As in [22–25] using prediction models (Neural Networks, long short-term memory (LSTM), Least Mean Square algorithm (LMS)) to reduce temperature, humidity, light, and voltage data sent from the sensors to the sink node in a wireless sensor network. In [26], authors proposed a prediction model to reduce the periodical information uploaded from smart industrial machines to the monitoring platform by not sending data of the same value or data increasing/decreasing linearly because it can be easily deduced by comparing the last two values received.

#### Based on a classifier

Data transmission reduction solutions can also be based on a classification model. Those models are trained on the importance of data according to the system, then used at the IoT object’s level to define if this data is worth sending or not. Such in [10] for wearable sensor networks or between mobile IoT devices and an edge server in [27].

#### Based on compression methods

Several papers use data compression to reduce the size of data sent from one entity to another. It consists of reducing the number of bits used to represent the message by applying a compression algorithm. Hu et al. proposed in [8] to use CNN for low power IoT devices to do content-aware and task-aware image compression before sending data to the cloud. Jarwan et al. in [23] used compression algorithms such as principal component analysis, non-negative matrix factorization and others to reduce the size of data sent from the cluster heads to the sink node (server, cloud). In [28], Uthayakumar et al. proposed a low complexity image compression schema for energy-constrained sensors in WSN. They proposed to use a specified algorithm (neighborhood correlation sequence (NCS)) before the compression algorithm. NCS performs bit reduction by generating the optimal (minimum number of bits) codeword for each pixel value.

#### Based on heuristic methods

Heuristic methods can also be used as in [29], authors proposed to send the first image captured by a camera, used in an agricultural context, to the cloud. Then, whenever a new image is captured, it will be divided into patches then compared (patch by patch) with the previous one. If the change is significant only the changed patch will be sent to the cloud. Otherwise, nothing will be sent. On the server’s side, the image is reconstructed using the previous image and the changed parts. For visual sensor networks, authors of [30] proposed to eliminate near-duplicate images using near-duplicate clustering, seed image selection then deleting the rest of images in the cluster. In [31], for systems like turning the light or the air conditioning on, where temperature, light, humidity data is sent to the cloud consistently. The authors proposed to use a machine learning model at the sensor level to calculate the number of humans in a room, then send it to the cloud server only if it is different than the previous transmission.

**Table 1:** Recent proposals for data transmission reduction

Data type	System	Transmission reduction method
**Based on prediction model**		
Temperature, Humidity, Light, Infrared, and Voltage	Sensor nodes and a central workstation	A synchronized prediction model on both sides, transmits only the values that surpasses a predefined error threshold [22]
Temperature, humidity, and light intensity	Between sensors and the cluster head	Both sides are using the same prediction model to predict the next data. If the prediction is different from the real captured data, the captured data will be sent to the cluster head node [23]
Humidity, temperature, light, and voltage values	Between a sensor node and the gateway in a WSN for environmental monitoring	The sensor and the gateway predict (using Least Mean Square algorithm) the future value simultaneously based on the past measurements [24]
Current production capacity, the volume of produced bottles, the volume of broken bottles, temperature and pressure	Between smart industrial machines and the server monitoring the system	If the new captured data is equal to the previous one or has a linear behavior, it can be deducted by the server [26]
Temperature, humidity, light, voltage values	Sensor nodes and cluster heads in a WSNs	Least-Mean-Square (LMS) algorithm to predict the next value. The sensor node sends the captured values until the cluster head is able to predict them with an acceptable error [25]
**Based on classifiers**		
Movement of the person	Wearable sensor networks (on a human body with a cluster head)	Classifiers (machine learning algorithms) that allow sensor nodes to decide if current sensor readings have to be transmitted to the cluster head or not [10]
Vehicles’ locations	Mobile IoT devices and an edge server	A machine learning model trained on the importance of data, each captured data is classified as important or not and only the data of importance in tra c flow prediction will be sent to the server [27]
**Based on data compression**		
Images	Between low power IoT devices and the cloud	Proposed content-awareness and task-awareness compression method using tiny machine learning models [8]
Temperature, humidity, and light intensity	Between cluster heads and the cloud	Data compression done at the cluster head to benefit from the spatio-temporal correlation of the data collected by the sensors of the cluster [23]
Images	visual sensors in WSN	Generating the optimal representation for each pixel value [28]
**Based on heuristics**		
Temperature, light, humidity	Application-specific IoT networks	Machine learning model in the sensor to calculate the number of humans in a room, send it to the cloud/edge server if it is different from the previous one [31]
Images	Visual sensor networks (camera nodes)	Eliminate near-duplicate images: near-duplicate clustering, seed image selection then delete all the rest images in the cluster [30]
Images	From a surveillance camera in an agricultural context to a server	Send the first captured image, then, whenever a change is detected only the changed part is sent. An image reconstruction phase is performed on the server’s side [29]

#### Findings

After reviewing works improving IoT systems’ performance we found that most of the solutions propose to improve one part of the system at a time: the network, the processing or the quality of data. Per contra, reducing the data sent improves the network functioning, the server’s responsiveness and saves the IoT device’s energy and resources. However, propositions in data transmission reduction are limited since most of them rely either on classifiers, compression or prediction methods (Table 1). Those methods take a long time to train, add heavy processing on the resource-limited object, and on top of that, they are not hundred percent error-free. Also, data transmission solutions focus on one IoT system, generally WSN containing temperature, humidity, and luminosity sensors since they generate numeric data that can be predicted easily. Moreover, for all these solutions, no generalization is proposed.

Therefore, our work is characterized by proposing a generalized (that can be projected on different IoT systems) and formalized (as we define mathematically all the functioning of the model) solution to reduce data offloaded from a connected device to the cloud. Thus, improving the system’s performance on several aspects.

In our previous paper [11], we described the problematic and the general idea of the proposed solution as well as evaluation metrics that permit to calculate the effectiveness of the solution. In this paper, we describe the proposed method more in detail. We also project it on different contexts by providing the methods can be used at each level of the solution for three different use cases.

### Section 3: data transmission reduction model

We propose a generalized formal data transmission reduction model independent of the system and the data type. This formalization is based on two main ideas:

• Not sending data until a significant change occurs.
• Sending a lighter size entity permitting the cloud to deduct the data captured by the IoT device without actually receiving it.

This section includes the general description of the solution as well as the mathematical representation of the model.

### General description

**Fig. 1:**
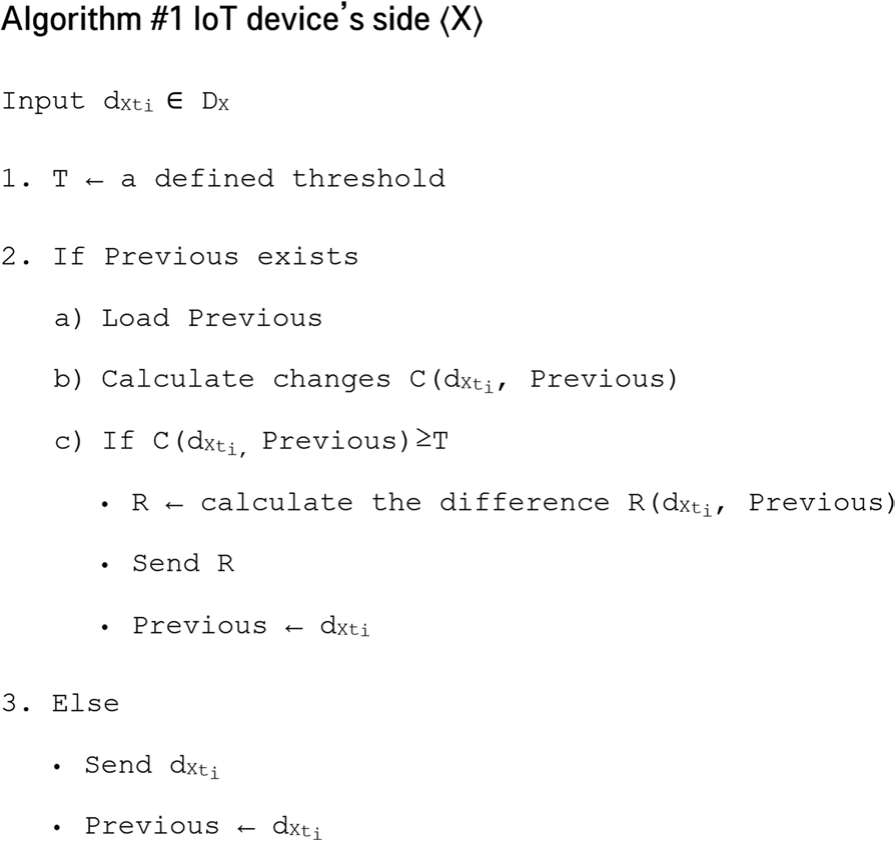
Operation algorithm at the connected object level

**Fig. 2:**
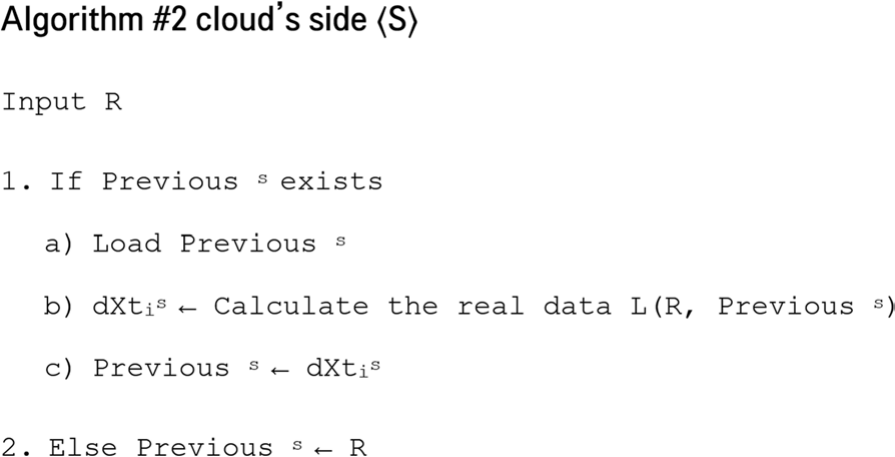
Operation algorithm at the cloud level

The proposed data transmission reduction model is represented by algorithm 1 and 2 (Figs. 1 and 2). For the connected device’s side (Fig. 1), when the data is captured, a test operation will take place *If Previous exists* which means that this is not the first data captured by this object. The previous data will be loaded *Load Previous* then the change between this new captured data and the previous one will be calculated $C\left({dx}_{t},Previous \right)$. If the calculated change value is significant, superior than the defined threshold *T*, $C\left({dx}_{t},Previous \right)$ ≥ *T*, the entity to be sent to the cloud is then calculated. This entity represents a relationship between the two data samples (the new data and the previous one) $R\left({dx}_{t},Previous \right)$.

If the change is smaller than the threshold *T*, *C*(*d*_*Xt*_*, Previous*) < *T* the new data will be ignored since it does not provide useful information for the cloud server.

In case of the first data captured by this device, data *d*_*Xt*_ will be directly transmitted to the cloud send *d*_*Xt*_ and stored as previous data on the device side *Previous ← d*_*Xt*_.

Regarding the server’s side (Fig. 2), when data is received from an object, if other data has been previously received from this object *If Previous*^*s*^* exists*, the previous data of this device (either received entirely or calculated at server level) is loaded *load Previous*^*s*^. Then, the previous data and the received entity are used to calculate the new data captured by the device L(R*, Previous*^*s*^).

Then, this calculated data d^*s*^*i* is stored on the server as previous data *Previous*^*s*^ so it can be used to calculate the next data.

In case of the first data, it will be just stored at the server *Previous*^*s*^* ← R*.

### Formalization

In this section we define mathematically the generalized methods composing this model. This will allow a fluid projection on different IoT system offloading data to a more powerful entity.

#### IoT object’s side:

• *X* is the connected device capturing data from the environment. This de vice can be a temperature sensor, a luminosity sensor, a proximity sensor, a surveillance camera, a smart phone or any end device in an IoT system.
• *S* is the cloud server to which the device *X* is connected. The computing capacities of the server are used to process the data received from the device *X.*
• *D*_*X*_ is the data collected by the smart device *X* from the moment it is connected to the IoT system until it leaves the system.
• *d*_*Xti*_ is the data captured by device *X* at time *t*_*i*_.
  $${D}_{x}=\left\{{d}_{{xt}_{0}},{d}_{{xt}_{1}},\dots ,{d}_{{xt}_{i}},{d}_{{xt}_{i+1}},\dots \right\}$$

We start from the principle that the data flow captured by the sensor is discrete and can be represented as a function of time.

• This discrete data is obtained by capturing data depending on the increment of time *t*. Time *t* is incremented each period of time ∆_*t*_*:*
  $${\Delta }_{t}={t}_{i+1}-{t}_{i}$$

The value of ∆_*t*_ is defined either at the implementation of the IoT system or at the injection of this device into the system.

• When new data ${d}_{{xt}_{i}}$ is captured, the device calculates the change between it and the previous data sent to the cloud Previous. This Previous data can be ${d}_{{xt}_{i-1}},{d}_{{xt}_{i-2}}$ or any data captured before as long as it’s the last one sent to the cloud.

If the change is important between the new captures data and the last update on the cloud, a sending will take place. This importance is defined by the threshold *T*. Otherwise, the new captured data ${d}_{{xt}_{i}}$ will be ignored. This concept is expressed as follows:

$$\left\{\begin{array}{c}If\;i=0\;send\;{d}_{{X}_{t0}}\\ Else\;if\;C{(d}_{{X}_{ti}} ,Previous)\ge T\;send\;R\;\equiv R{(d}_{{X}_{ti}} ,Previous)\\ Otherwise\;no\;sending\;is\;performed.\end{array}\right.$$

• $C\left({d}_{{Xt}_{i}},\mathrm{Previous}\right)$ is a function calculating the difference between data collected by device *X* at time *t*_*i*_ and the last data sent to the cloud Previous. The function *C* (.,.) takes as input two data instances of the same nature and sufficiently similar. This function can be a simple subtraction, a dissimilarity measurement method, a change detection method or other. The choice of this function depends on the nature of data manipulated, nature and capacities of the IoT device and the main goal of the IoT system.
• *T* is a threshold representing the lower barrier of the difference *C* (*d*_*Xti*_, Previous) to consider the existence of change between *d*_*Xti*_ and the last data sent. *T* is of the same nature as the output of *C* (*d*_*Xti*_, Previous) whatever it was (real number, percentage, vector, matrix, etc.). This threshold can be defined using a thresholding method after adapting it to the nature of the problem being treated.
• *R* (*d*_*Xti*_, Previous) is the method used to calculate the entity to be sent to the cloud when the difference exceeds the threshold. In common cloud-based IoT systems.

*R* (*d*_*Xti*_, Previous) = *d*_*Xti*_ which means that when a change occurs the new captured data *d*_*Xti*_ is sent directly to the cloud. The solution we are proposing, consist on sending a relationship between *d*_*Xti*_ and Previous to the cloud that must satisfy the following condition:

$$\mathrm{Size}\left(R\right)<\mathrm{Size}\left(d_{{xt}_i}\right)$$

Where Size(.) is a function calculating the size of data in bytes. This condition assures the efficiency of this new solution sending less data via the network *N* and therefore improving latency and bandwidth consumption.

*R* can be the changing part of data, the difference *C* (*d*_*Xti*_, Previous), the rate of change or others.

#### Cloud server’s side:

On the cloud server’s side, data is calculated as follow:

$$\left\{\begin{array}{c}{d}_{{X}_{t0}}^{s}= {d}_{{X}_{t0}}\\ {d}_{{X}_{ti}}^{s}=L\left(R,{Previous}^{s}\right)\end{array}\right.$$

• ${d}_{{Xt}_{i}}^{s}$ is the data captured by the device *X* at time *t*_*i*_ calculated on the server’s side.
•${Previous}^{s}$ is the previous data received calculated on the server’s side.
• For $i=0:{d}_{{Xt}_{0}}^{s}={d}_{{Xt}_{0}}$ because it is not calculated on the cloud server’s side but received entirely from the smart device.
• $\mathrm{L}\left(\mathrm{R},{\mathrm{Previous}}^{\mathrm{s}}\right)$ Calculates data captured by the device on the server’s side using the relationship R ≡ R (dXti, Previous) received from the object and the previous data calculated by the server Previouss. This precedent data was calculated by the server at the previous step using the same formula.

The efficiency of the data calculation operation on the server is evaluated by the following condition:

$${C}^{s}\left({d}_{{X}_{ti}}^{s}{,d}_{{X}_{ti}}\right)<\varepsilon$$

Where:

• *C*^*s*^(*.,.*) is a function that calculates the difference between data captured by the device and the same data calculated by the server. It can be the same function used to calculate the difference on the IoT device’s side: ${C}^{s}\left(.,.\right)=C\left(.,.\right)$
• *ε* is a threshold that needs to be defined. This condition assures that the calculated data ${d}_{{Xt}_{i}}^{s}$ is similar enough to the real data ${d}_{{Xt}_{i}}$ so as not to affect the data processing and the decision making performed at the cloud level.

The introduction of this generalization makes it possible to model, evaluate and solve IoT systems’ problems in batches instead of treating each system individually. Unlike system-dependent solutions, where no solutions are proposed for complex and special systems, using our model, it suffices to project on the use case to reduce the data sent. Moreover, there is no need for collecting a dataset or prior learning phases.

### Conditions of use of the model

The defined model is generalized and can be applied to different systems however it will give better results if the following conditions are met:

- Data is captured in one site and processed in another.
- The system is capturing discrete data successively and intensely.
- The type of data captured is heavy and consumes a lot of time and bandwidth to be sent via the network.
- The object capturing data is of limited resources.
- Time is a critical parameter for the efficiency of the reaction.

Those characteristics are common for most of the IoT systems such as in smart homes, security cameras, sensor monitoring in industrial field, and cloud gaming, etc.

### Section 4: use cases

To prove the applicability of the proposed model on systems that falls under the overmentioned characteristics, in this section, we will provide the projection and the methods used to apply this model on several real-world use cases.

#### Traffic camera

Previously, traffic control systems were basic, a camera recording videos and a traffic controller (a policeman) in the control station receiving and analyzing those videos. This method is not only time-consuming and requires a large staff but also subjective, inaccurate and provides incomplete results [32]. Nowadays, traffic monitoring is way beyond that. Traffic cameras are placed everywhere in the roads, and data processing is done by AI models either in the camera or in the cloud. Achieving different tasks such as measuring the traffic flow, detecting accidents, traffic light violations, speed limits exceeding, plate numbers of passing cars, etc.

#### Characteristics of traffic monitoring system

• Traffic cameras capture a huge amount of data.
• The transferred data in this system is images which consume more bandwidth, resources and time compared to numeric data.
• Several situations of non-change occur in this IoT system such as empty road or the same traffic congestion.
• Cameras used in traffic controlling have limited resources.
• Tasks of traffic monitoring and laws violations detection are resource consuming (image processing, object tracking, etc.).
• Strong processing resources lead to a better effectiveness of results.
• There are 200 million surveillance cameras in the roads (2019 statistics) [33], and reducing spending of unnecessary data will improve the effectiveness of the monitoring system.
• The camera is fixed in a road, therefore there are similarities between previous and present data that our solution relies on to calculate the changes.

#### Projection of the proposed model on the use case

In this section we will present the projection of our proposed model on traffic monitoring system by giving the corresponding function for each one of the abstract methods.

#### Camera level

• C (d_Xti_,Previous): To compare the new captured image to the previous one we can use Structural Similarity Index (SSIM). This metric was first introduced by Wang et al. [34] to calculate image quality degradation after compression or transmission. After popularization, it became a standard for changes quantification between two images in different contexts such as remote sensing for satellite images, surveillance cameras, object tracking, etc.

For two images *x* and *y*, SSIM is calculated as follows:

1 $$\begin{array}{c}SSIM\left(x,y\right)=\frac{\left(2{\mu }_{x}{\mu }_{y}+{c}_{1}\right)\left(2 {\sigma }_{xy }+{c}_{2}\right)}{\left({\mu }_{x}^{2}+{\mu }_{y}^{2}+{c}_{1}\right)\left({\sigma }_{x}^{2}+{\sigma }_{y}^{2}+{c}_{2}\right)}\end{array}$$

Where:

µ_x_ is the average of *x*

µ_y_ is the average of *y*

*σ*^2^is the variance of *x*

*σ*^2^ is the variance of *y*

*σ*_*xy*_ is the covariance of *x* and *y*

*c*_1_ and *c*_2_ are specific variables used to stabilize the division.

The SSIM value vary between − 1 and 1, where a value of 1 indicates perfect similarity and a value of − 1 indicates that the two images are totally different.

• R: After detecting the change, the next step is to calculate the entity to be sent to the cloud. In this use case, the changed parts of the image are the best option since they are of lighter size compared to the original image and they spot the most important information. To extract the changed parts a threshold method is applied to the difference image calculated using SSIM. One effective thresholding algorithm that can be used in this situation is Otsu’s thresholding algorithm. The goal of this algorithm is to find the optimum value *t* separating image pixels into two classes: foreground and background or important and not important [35]:

2 $${\sigma }_{w}^{2}\left(t\right)={w}_{1}\left(t\right){\sigma }_{1}^{2}\left(t\right)+{w}_{2}\left(t\right){\sigma }_{2}^{2}\left(t\right)$$

*w*_1_(*t*) and *w*_2_(*t*) are the probabilities of the two classes.

The objective is to minimize the variance *σ*^2^ (*t*) to define the optimal *t* dividing the two classes. ${\sigma }_{1}^{2}$ and ${\sigma }_{2}^{2}$ are the variances of these classes.

T: In this use instead of using a threshold to detect if $C\left({d}_{{X}_{ti+1}}{,d}_{{X}_{ti}}\right)$ is important, we prefer evaluating if the change is worth sending by part of image. That means that instead of deciding if this new image worth sending we decide if the parts of this image worth sending which allows better sending precision.

**Fig. 3:**
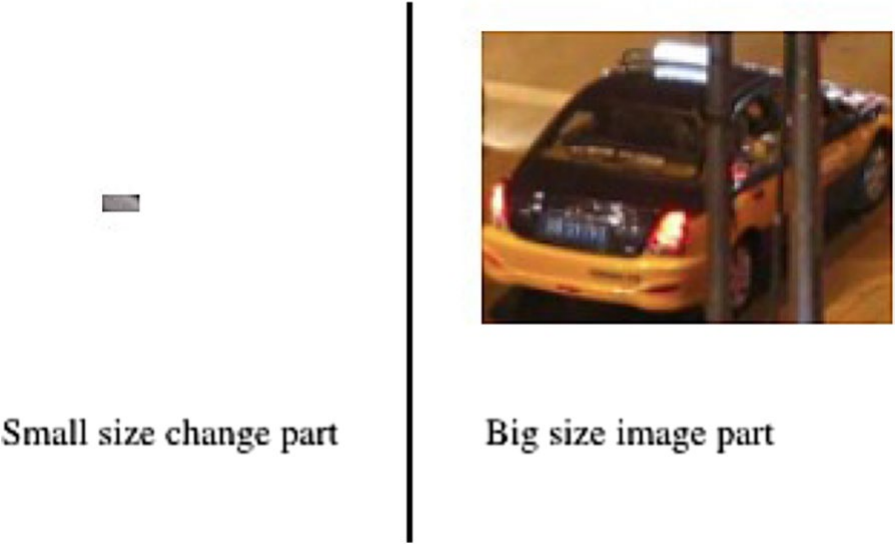
Example of small changes that can be ignored compared to change sent to the cloud

After observation on extracted change parts between successive images, we deduced that there is a direct proportion between the size of the image part and its importance. Small size change portions are just dots or lines that do not represent any information. Figure 3 shows an example of extracted change parts between two successive images where two kinds of change can be detected: big size parts that are most of the time informative and small size parts that do not usually provide any information. Therefore, the portion’s size can be used as an importance filter.

#### Cloud level

- L (R, Previous ^s^): At the cloud level the objective is to detect the cars and congestions. Object detection models are generally composed of two steps: generating region proposals from the image then classifying them. In this case, we have already detected the regions of interests (regions containing changes) at camera level. Therefore, image parts can be classified using the second part of the object detection algorithm.

**Fig. 4:**
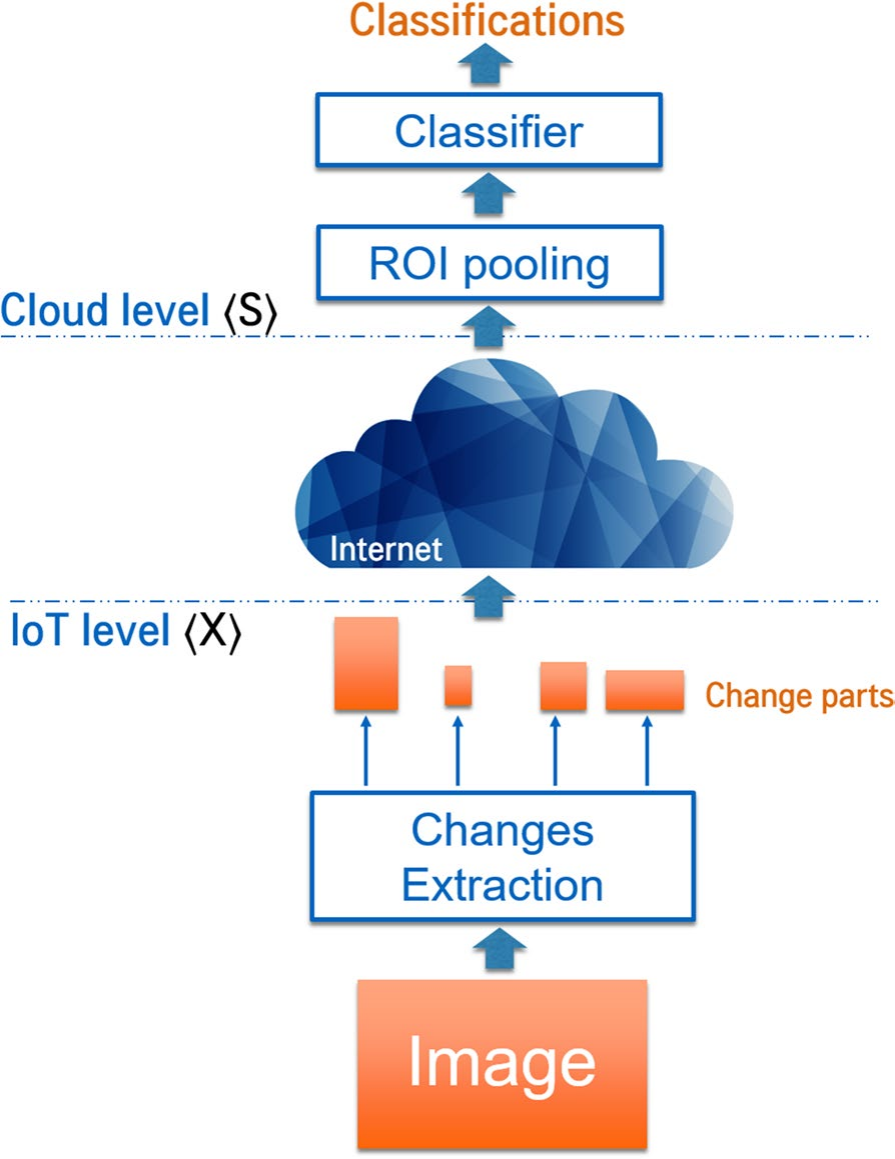
Faster R-CNN partitioning to fit our proposed model

Faster R-CNN is a perfect match for our needs since, besides being effective for real-time object detection, it consists of two parts: a CNN for region proposal and another CNN to classify those regions. Those two parts can be easily separated and executed in different locations. Furthermore, the Region of Interest Pooling (RoI) Layer used between the two parts will solve the problem of the multi-sized image parts that we are sending via the network and make them fit the input size of the classifier. Figure 4 represents how Faster R-CNN can be divided to apply our model.

Thus, the work done at the cloud level in this use case is equivalent to use the second part of a pre-trained Faster RCNN to classify detected image parts without calculating the real captured image.

### LiDAR sensors

Light Detection and Ranging (LiDAR) is a sensor measuring the time-of-flight of light. It targets an object with a laser then measures the time for the reflected light to return.

Data generated by this sensor is called point cloud data: a group of points representing objects or space in three-dimensions. Each point represents a single light measurement, stitched together, they create a complete point image of a scene [36]. LiDAR sensors are widely used for self-driving cars to sense the surroundings since it can provide 360^*◦*^ information with a detection range of 120 m. It is used to detect vehicles, pedestrians and obstacles and the distance between the detected object and the car [37].

Due to the considerable volume of point cloud data, its transmission consumes a lot of time and resources [38]. The use of our data reduction model will help accelerating and improving the functioning of the system.

Projection of the proposed model on the use case:

- LiDAR sensor’s level
  - C (d_Xti_, Previous): Several methods exist to compare point cloud data [39]: Cloud to Cloud (C2C), Multiple Model to Model Cloud Comparison (M3C2), Cloud to Mesh (C2M), DEM of difference (DOD).
    Those methods are generally used for aerial remote sensing to detect changes in buildings and landscape. In our case we can use them to calculate the difference between two data instances captured by a LiDAR sensor.
    Even if a little less efficient, C2C is the simplest and fastest method to calculate changes between two point clouds [40] which suits the specification of our system.
    The result of calculating C2C between two cloud points is the set of differences between the point in the first point cloud and the nearest neighbor in the second.
  - T: To extract only important changes, the value of this difference need to be thresholded since values close 0 means a point coincidence (no significant change occurred).
    Root Mean Square Error (RMSE) is generally used to establish the minimum level of change detection between point clouds [39, 41]. It is defined as follow:
    3 $$RMSE=\sqrt{\frac{{\sum }_{j=1}^{j=n}{\left({d}_{{X}_{ti-1}}\left(j\right)-{d}_{{X}_{ti}}\left(j\right)\right)}^{2}}{n}}$$
  - R: Since one point in LiDAR data is represented by three coordinates that are heavy data to send via the network, we send instead the distance calculated by the C2C ( $C\left({d}_{{X}_{ti+1}}{,d}_{{X}_{ti}}\right)$) method. Previous point and distance with the new one are enough to calculate the data.
    Furthermore, we will only send distances considered important (higher than the threshold) and thus we can achieve an important data reduction.
- Cloud level
  - L (R*,*Previous ^s^): Real data at cloud level will be calculated by finding the position of all points by adding the distance received to its nearest point in previous data stored in the server. When no distance is received the coordinates of this point in this new data are the same as the previous.

#### Health wearables

Remote patient monitoring is a system where a smartphone or a wearable device (smart watch, smart glasses, smart clothing, etc.) is capturing health metrics and sending it to the cloud server used by the hospital. The received data is analyzed and projected on patient’s medical records in order to recommend treatments or generate alerts so that healthcare professionals can intervene [42, 43]. This IoT system exempts the patient from traveling to the hospital (especially for the period of the Covid-19 pandemic), collecting metrics by himself and the most important provides rapid intervention in case of danger [44]. However, the volume of data exchanged between the connected device and the server is hard to manage [10]. Since non-changed data doesn’t provide any additional information for such a system, our proposed model will ensure a significant reduction without performance degradation.

Wearables and smartphones provide a diversity of sensors that can be used in this context such as accelerometer (detecting steps or motion), heart rate sensor (detects heart bits per minute), body and environment temperature sensors, pH sensor, blood oxygen sensor, gyroscope (detecting head movements), etc.[44].

Projection of the proposed model on the use case:

- Wearable level
  - C (d_Xti_*,*Previous): Sensors used in remote health monitoring capture real numbers as data type: temperature, pH value, oxygen level,etc. Change detection between two data values is performed by calculating the absolute difference between pair of data for each sensor in the wearable device.
  - T: To reduce data sending furthermore, we can compare the new captured data with the normal value of this health metric such as the normal corporal temperature between 36*.*1^*◦*^C and 37*.*8^*◦*^C and normal heart beat between 60 and 100. This way we will be sure that something is happening and avoid sending data when the change is normal. This can work for most sensors except for those capturing position or rotation.

Also we can compare the captured value with the normal value for this person because it may be different than the standard. For example, athletes might have a normal heart rate closer to 40 beats per minute [45]. This way we significantly reduce sendings for special categories.

- R: In this IoT system, the main problem is not the size of data but density of sending. Since we significantly reduced the amount of data to be sent, sending the new captured data in situations of significant change won’t consume much resources since it is one numeric data.

- Cloud level
  - L (R, Previous ^s^) In this case, data received by the cloud is directly processed since it is the same data captured by the wearable sensors. However, the significantly reduced sending density permits not flooding the server with data that is generally non-significant and therefore speeding up the response time and ameliorating the system’s performance.

In this section we presented the projection of our general model on several IoT systems, image data from a traffic camera, point clouds from LiDAR sensors and numeric metrics captured by wearable sensors. We provided the corresponding of each function of the model proving the possibility of projecting our model on all the systems capturing and sending data that respect the conditions previously mentioned.

### Section 5: conclusions

In this paper, we proposed a generalized formal model to reduce data transmitted from IoT objects to the cloud for processing. We defined mathematically the abstracted methods ensuring the proper functioning of the model as well as the generalized evaluation metrics. This model frames the operation of reducing data sending and therefore improves object-cloud communication for a bulk of IoT systems not having to treat each case separately anymore. We also provided projections on real life IoT use cases by giving the functions that can be used to apply the model on this system.

To our knowledge, this work is the first proposing such a generalization and abstraction for data transmission reduction as well as projections on IoT systems of different types. For future work, we will be studying the possibility of using the same method to calculate the difference between data of different types. This way we add another generalization aspect to the proposed model permitting a more fluent data transmission reduction.

## Data Availability

Not applicable.
